# Molecular Survey on A, B, C and New Avian Metapneumovirus (aMPV) Subtypes in Wild Birds of Northern-Central Italy

**DOI:** 10.3390/vetsci9070373

**Published:** 2022-07-20

**Authors:** Claudia Maria Tucciarone, Giovanni Franzo, Matteo Legnardi, Daniela Pasotto, Caterina Lupini, Elena Catelli, Giulia Quaglia, Giulia Graziosi, Emanuela Dal Molin, Federica Gobbo, Mattia Cecchinato

**Affiliations:** 1 Department of Animal Medicine, Production and Health (MAPS), University of Padua, Viale dell’Università 16, 35020 Legnaro, Italy; claudiamaria.tucciarone@unipd.it (C.M.T.); giovanni.franzo@unipd.it (G.F.); daniela.pasotto@unipd.it (D.P.); mattia.cecchinato@unipd.it (M.C.); 2 Department of Veterinary Medical Sciences, University of Bologna, Via Tolara di Sopra 43, 40064 Ozzano dell’Emilia, Italy; caterina.lupini@unibo.it (C.L.); elena.catelli@unibo.it (E.C.); giulia.quaglia2@unibo.it (G.Q.); giulia.graziosi2@unibo.it (G.G.); 3 Istituto Zooprofilattico Sperimentale delle Venezie, Viale dell’Università 10, 35020 Legnaro, Italy; ema90dalmolin@gmail.com (E.D.M.); fgobbo@izsvenezie.it (F.G.); 4 Department of Comparative Biomedicine and Food Science (BCA), University of Padua 16, Viale dell’Università, 35020 Legnaro, Italy

**Keywords:** avian metapneumovirus, subtypes, wild birds, mallard, molecular epidemiology

## Abstract

**Simple Summary:**

Avian metapneumovirus (aMPV) is a common pathogen in poultry and has been detected in wild birds, suggesting the possible role in viral dissemination. A feature of aMPV is its genetic and antigenic variability, which has allowed the identification of various subtypes of the virus with different characteristics in terms of host tropism. Two new subtypes of aMPV were recently identified in gulls and parakeets. We aimed to explore the epidemiology of old and new aMPV subtypes in wild birds. Samples were collected in Italy during the surveillance of avian influenza in wild species and were tested with two multiplex real time RT-PCRs that were able to detect and distinguish the aMPV subtypes (A, B, C, gull, and parakeet subtypes). All of the individuals were negative, except for one mallard that was positive for aMPV subtype C. The M and G genes of this strain were molecularly characterized and revealed similarities with Chinese and European strains, including an Italian sequence that was previously detected in a widgeon. These findings confirm the susceptibility of mallards, which are closely related to domestic species, highlighting the importance of the epidemiological monitoring of aMPV circulation.

**Abstract:**

Recent insights into the genetic and antigenic variability of avian metapneumovirus (aMPV), including the discovery of two new subtypes, have renewed interest in this virus. aMPV causes a well-known respiratory disease in poultry. Domestic species show different susceptibility to aMPV subtypes, whereas sporadic detections in wild birds have revealed links between epidemiology and migration routes. To explore the epidemiology of aMPV in wild species, a molecular survey was conducted on samples that were collected from wild birds during avian influenza surveillance activity in Italy. The samples were screened in pools by multiplex real time RT-PCR assays in order to detect and differentiate subtypes A, B, C, and those that have been newly identified. All the birds were negative, except for a mallard (*Anas platyrhynchos*) that was positive for aMPV subtype C (sampled in Padua, in the Veneto region, in 2018). The sequencing of partial M and full G genes placed the strain in an intermediate position between European and Chinese clusters. The absence of subtypes A and B supports the negligible role of wild birds, whereas subtype C detection follows previous serological and molecular identifications in Italy. Subtype C circulation in domestic and wild populations emphasizes the importance of molecular test development and adoption to allow the prompt detection of this likely emerging subtype.

## 1. Introduction

Initially identified in South Africa in 1978 [1], avian metapneumovirus (aMPV) is a pathogen that primarily infects turkeys and chickens [2]. aMPV infection alone is mainly responsible for respiratory disease in poultry, with high morbidity but contained mortality [3]. The coinfection between aMPV and *Escherichia coli* has been associated with swollen head syndrome (SHS) [3] in chickens, which shows swelling of the periorbital and infraorbital sinuses [4]. Reproductive performances and egg quality may also be affected [5,6].

Similar to many other RNA viruses, aMPV displays significant genetic heterogeneity [7], and different subtypes have been distinguished based on antigenic [8] and genetic features [9]. A and B subtypes were the first subtypes recognized [9]. These subtypes spread worldwide and reached Europe in the 1980s [10,11]. A third subtype, aMPV subtype C, was initially identified in the US [12] in turkeys and then in wild birds [13,14]. A second and distinct lineage of this subtype was detected in Europe [15], although this subtype showed a tropism for ducks rather than turkeys. A fourth subtype (aMPV-D) was detected only in France in archival samples from turkeys [16].

All aMPV subtypes proved to infect *Galliformes* in experimental conditions [17], even though there were some differences in susceptibility, clinical sign development, and shedding. Turkeys appeared to be susceptible and capable of transmitting all four subtypes, except for the aMPV-C subtype of duck lineage. Chickens appeared to be fully susceptible to subtype B, and they seroconverted without shedding subtype A, subtype C of turkey lineage, and subtype C of duck lineage in the absence of clinical signs. Ducks hosted viral replication and showed clinical signs only when challenged with the aMPV-C subtype of duck lineage [17].

The differences in host tropism fit well with the current epidemiological situation, where aMPV-A is encountered less and less frequently in reared poultry [18,19] (probably due to the lower shedding ability of chickens [17]), aMPV-B is widely present and tends to cluster both geographically and temporally [18,19], and aMPV-C continues to circulate in the US in both domestic [20] and wild [14] populations, whereas few detections were made in France [15], the Netherlands [21], Italy [22], South Korea [23], and China [24,25].

With the exception of aMPV-D, aMPV subtypes have also been identified in various wild species. aMPV subtype A has been detected in wood ducks (*Aix sponsa*), mandarin ducks (*Aix galericulata*), white-faced whistling ducks (*Dendrocygna viduata*), rock pigeons (*Columba livia*), American kestrels (*Falco sparverius*), white-eyed parakeets (*Psittacara leucophthalma*) [26], white-cheeked pintails (*Anas bahamensis*), rusty-margined guans (*Penelope superciliaris*), and Orinoco geese (*Neochen jubata*) [27]. aMPV subtype B has been detected in white-cheeked pintails, white-faced whistling ducks, and rock pigeons [27].

aMPV subtype C has been found in mallards (*Anas plantyrhynchos*), greylag geese (*Anser anser*), and common gulls (*Larus canus*) in Europe [21], and in American black ducks (*Anas rubripes*), American wigeons (*Mareca americana*), blue-winged teals (*Spatula discors*), Northern shovelers (*Spatula clypeata*), mallards, wood ducks, Canadian geese (*Branta canadensis*), English sparrows (*Passer domesticus*), barn swallows (*Hirundo rustica*), and European starlings (*Sturnus vulgaris*) in North America [14,28,29].

Antibodies against aMPV have been identified in sea gulls (*Larus argentatus argentatus*) in Germany [30], and in American coots (*Fulica americana*), American crows (*Corvus brachyrhynchos*), Canadian geese, cattle egrets (*Bubulcus ibis*), and rock pigeons in the US, where coots and geese were found to be positive for subtype C by direct detection [14].

Recently, two new aMPV subtypes were discovered by deep sequencing techniques in a great black-backed gull (*Larus marinus*) [31] and a monk parakeet (*Myiopsitta monachus*) [32]. These new viruses seem to be intermediate subtypes between the cluster of aMPV subtypes A, B, and D and the cluster of aMPV subtype C and subtypes A and B of human Metapneumovirus (hMPV) [32].

The variety of genetic and biological features of aMPV, as well as its broad host range, prompted the present study. This study aimed to investigate the presence of the currently circulating aMPV subtypes (A, B, C) and those that have been newly discovered in wild birds in Northern Italy in order to explore the viral presence and possible viral flux between domestic and wild populations.

## 2. Materials and Methods

### 2.1. Sample and Data Collection

Samples were collected during the passive and active avian influenza surveillance activity that was performed by the Istituto Zooprofilatico Sperimentale delle Venezie (IZSVe) (Legnaro, Padua). During active surveillance, birds were trapped and sampled mainly by tracheal or oropharyngeal swab collection, whereas the organs from dead birds were collected during passive surveillance.

The samples were processed for nucleic acid extraction with the QIAsymphony DSP Virus/Pathogen Midi kit (Qiagen, Hilden, Germany), in combination with the automated system QIAsymphony SP (Qiagen, Hilden, Germany). Plates containing the extracted samples were then stored at −80 °C until further processing.

The samples that were negative for avian influenza were delivered to the Laboratory of Infectious Diseases at the Department of Animal Medicine Production and Health (MAPS) (Legnaro, Padua) at the University of Padua, together with the available information about the species, age, matrix, date, and place of collection.

The minimum sample size was preliminarily determined to detect at least one positive sample with 95% confidence assuming an infinite population, a test sensitivity of 90%, and an expected prevalence lower than 0.5% at the individual level (http://epitools.ausvet.com.au, accessed on 1 May 2022).

A database was organized to record the signalment of the animal, and the identification, plate number, and position of each sample. The extracted samples were assigned to and mixed in pools of a maximum of 8 individuals, following the sample order in the plates.

### 2.2. Molecular Analyses

The pools were tested using a specific multiplex real time RT-PCR for A and B subtypes and a multiplex real time RT-PCR designed to detect both subtype C and the new subtypes identified in gulls and parakeets. The primers and probes that were used are reported in Table 1. Real time RT-PCR reactions were performed using a SuperScript^®^ III One-Step RT-PCR System with a Platinum^®^ Taq DNA Polymerase kit (Invitrogen™, Waltham, MA, USA) on a LightCycler^®^ 96 Instrument (Roche, Basel, Switzerland). We added 2 μL of RNA template to the following mix: 5 μL of 2× Reaction Mix, 0.2 μL of SuperScript™ III RT/Platinum™ Taq Mix, 0.8 μM of each primer for C, gull, and parakeet subtypes and 0.5 μM of each primer for A and B subtypes, 0.25 μM of each probe for C, gull, and parakeet subtypes, and 0.3 μM of each probe for A and B subtypes. Ultrapure molecular biology water was added up to a volume of 10 μL. The thermal protocols for amplification included a reverse transcription phase at 50 °C for 15 min and a 2-min-long activation phase at 95 °C, followed by 55 cycles of denaturation at 95 °C for 5 s and annealing/extension at 60 °C for 30 s for C, gull, and parakeet subtype detection and 20 s for A and B subtype detection.

The assays were validated using ten-fold serial dilutions of a plasmid containing the target sequences of A (Acc. Num. MF093139), B (Acc. Num. JF810662), C (Acc. Num. HG934338), gull (Acc. Num. MN175553), and parakeet (Acc. Num. MK491499) subtypes. The assays showed a limit of detection (LoD) of 10^0^ copies/µL and an efficiency of 2.06 for subtype A, 1.90 for subtype B, 2.05 for subtype C, 2.19 for the subtype detected in gulls, and 2.14 for the subtype detected in parakeets.

Each sample from the positive pools was tested again singularly using the same methods in order to identify and confirm the positive individuals.

The G gene of positive samples for A and B subtypes was amplified as previously described by Cecchinato et al., (2010) [33] in order to sequence and characterize the strains. The partial M gene of the samples that were positive for subtype C was amplified as described by Shin et al., (2000) [34], whereas the full G gene was amplified as described by Graziosi et al., (2022) [22]. The samples that were positive for the new subtypes detected in gulls and parakeets were tentatively amplified in the N gene, as described by Bayon-Auboyer et al., (1999) [35]. RT-PCRs were performed using a SuperScript™ III One-Step RT-PCR System with a Platinum™ Taq DNA Polymerase kit (Invitrogen™, Waltham, MA, USA) on an Applied Biosystems 2720 Thermal Cycler (Applied Biosystems, Waltham, MA, USA). The amplicons were then Sanger sequenced with the respective primer pair in both directions at Macrogen Spain (Madrid, Spain).

### 2.3. Phylogenetic Analyses

The chromatograms were visually inspected for a preliminary quality check using FinchTV software (Geospiza Inc., Seattle, WA, USA), and consensus sequences were assembled using ChromasPro 2.1.8 software (Technelysium Pty Ltd., Helensvale, QLD, Australia). The sequences were preliminary evaluated by BLAST search. Then, a database of available sequences was downloaded from GenBank and aligned to the sequences obtained from MEGA X [36]. The sequences were phylogenetically analyzed by reconstructing a Maximum Likelihood phylogenetic tree using MEGA X software [36] after downloading a database of the available sequences of the relative subtypes in addition to a reference sequence for all other subtypes (including human Metapneumovirus) from Genbank (Supplementary Tables S1 and S2). Branch support was calculated by performing 1000 bootstrap replicates, and bootstrap values ≥70% were considered reliable. The substitution model was selected based on the lowest Bayesian information criterion (BIC), calculated using MEGA X software [36].

## 3. Results

The sampling activity took place from 2018 to 2021 and a total of 1932 wild birds were sampled: 866 birds were sampled in 2018, 582 in 2019, 413 in 2020, and 71 samples in 2021. Sample collection was performed in the provinces of Bolzano (10), Ferrara (47), Pisa (52), Padua (825), Rovigo (713), Treviso (19), Venice (208), Verona (28), and Vicenza (30). Tracheal (1761), oropharyngeal (159), and conjunctival (1) swabs were used for the study, in addition to samples of lung tissue from dead birds (11). The species that were sampled are reported in Table 2.

The extracted samples were assembled into 262 pools, composed of a maximum of 8 samples each (mean 7.4). All of the samples tested negative for aMPV subtype A, B, and for the newly identified subtypes in gulls and parakeets. This allowed the exclusion of a prevalence higher than 0.15% with a confidence of 95% in the wild population, assuming a population size greater than 100,000 individuals. One tracheal swab of an adult mallard (1/862), negative for avian influenza virus (AIV) and sampled in 2018 in the Veneto region of Padua, was positive for aMPV subtype C, yielding an estimated prevalence of 0.12% (0.00–0.34%, IC95%) in the mallard population. All other species tested negative for aMPV-C, allowing the exclusion of a prevalence higher than 0.28% (IC 95%) in the remaining wild population with an estimated population size greater than 100,000 individuals.

**Figure 1:**
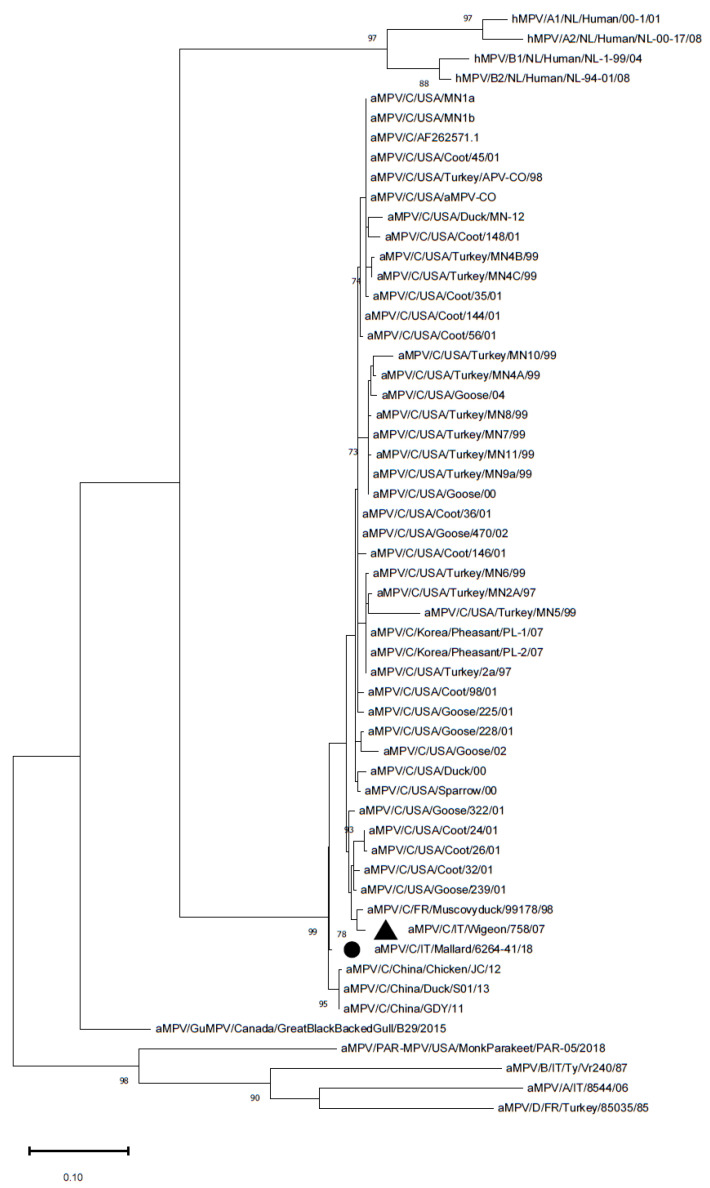
Phylogenetic tree reconstructed using the M gene of aMPV strains from Supplementary Table S1. The Italian mallard strain is marked with a black circle, whereas the previously detected Italian strain is marked with a black triangle. The tree was reconstructed using the Maximum Likelihood method and Tamura 3-parameter model with discrete Gamma distribution. Bootstrap support (>70%) is shown next to the branches. The tree is drawn to scale, with branch lengths measured in the number of substitutions per site. This analysis involved 56 nucleotide sequences. All of the positions containing gaps and missing data were eliminated. The final dataset was composed of 358 positions.

The aMPV-C strain was sequenced, yielding the partial sequence of the M gene and the complete sequence of the G gene, which were deposited in Genbank (Accession numbers ON457994–ON457995). The partial M gene sequence (359 nucleotides) was aligned to a database of 55 sequences that was downloaded from GenBank and/or was previously obtained (Supplementary Table S1). The phylogenetic analysis of the partial M gene showed that the aMPV-C strains detected in this study were placed in an intermediate position between the European lineage and Chinese cluster (Figure 1) (*p*-distance with the Eurasian wigeon strain: 0.03; mean *p*-distance with the Chinese clade: 0.01; mean *p*-distance with the clade containing the French and Italian strains: 0.03).

**Figure 2:**
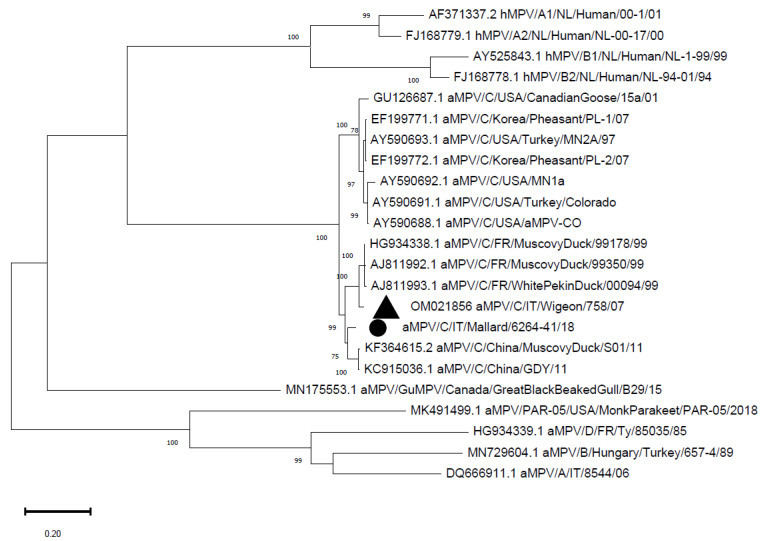
Phylogenetic tree reconstructed using the G gene of aMPV strains from Supplementary Table S2. The Italian mallard strain is marked with a black circle, whereas the previously detected Italian strain is marked with a black triangle. The tree was reconstructed using the Maximum Likelihood method and Hasegawa-Kishino-Yano model with discrete Gamma distribution (G) and a proportion of invariable sites (I). Bootstrap support (>70%) is shown next to the branches. This analysis involved 23 nucleotide sequences. All of the positions with less than 95% site coverage were eliminated. The final dataset was composed of 502 positions.

The complete G gene was 1758 nucleotides long. The sequence was aligned to a database of 22 sequences that was downloaded from GenBank and/or was previously obtained (Supplementary Table S2), and the above-mentioned clustering was confirmed (Figure 2) (*p*-distance with the Eurasian wigeon strain: 0.07; mean *p*-distance with the Chinese clade: 0.04; mean *p*-distance with the clade containing the French and Italian strains: 0.07).

## 4. Discussion

The present study examined a very large number of wild birds and benefitted from the annual avian influenza surveillance activity executed in Italy. With the exception of mallards, a greylag goose, a European starling, and a cattle egret, most of the sampled species have not yet been reported in the literature as aMPV hosts. The convenience nature of the sampling prevented the selection of target species for aMPV investigation. However, the majority of the species belong to the *Anatidae* family, which can be considered possible hosts of aMPV. Moreover, the species studied and their abundancy reflect the wild bird population of Northern Italy, with a focus on waterfowl wintering in key areas for the epidemiological monitoring of relevant pathogens at the domestic and wildlife interface [37].

Conversely, the recent discovery of new subtypes in gulls [31] and parakeets [32] testifies the importance of the wide monitoring of different species as aMPV reservoirs. As a matter of fact, evidence of sea gull susceptibility has already been proposed by Heffels-Redmann et al., (1998) [30] using serological means, although without identification of the responsible subtype. Unfortunately, no *Charadriiformes* or *Psittaciformes* were sampled in this study, likely explaining the lack of detection of the new subtypes since, in the case of a newly emerging subtype, it may be expected that circulation is limited to the original hosts. Nevertheless, the real impact of the infection sustained by the new subtypes, and the extent of their host range and circulation, have not yet been established. Therefore, it is necessary to gather more data about the possible diffusion of the new subtypes among other species in order to understand their origin and epidemiology.

The absence of A and B subtype detection is reassuring for the Italian poultry sector, which was recently shown to be profoundly vulnerable to the wild–domestic interface during the last HPAI epidemic [38]. It is likely that the low density of the wild bird population, together with the limited susceptibility and shedding ability of subtypes existing outside of their original host [17], hinders the circulation of A and B subtypes, thus containing the risk of spillover into the domestic population.

On the other hand, a recent study [39] reported the seropositivity for aMPV-C of an entire mallard flock reared in Lombardy (Northern Italy) during a serological survey on ducks and mallards at slaughter. Our direct identification further supports the susceptibility of this species and suggests that there is a precise connection between wild and domestic populations, especially since migrating birds may have frequent contact with resident urban birds in peri-urban and farming areas [40]. Moreover, mallards are abundantly reared in Central and Northern Italy for meat consumption and are released for hunting [41], so further investigations are needed to establish the possible links and the directionality of pathogen exchange between domestic and wild populations. Even though studies have shown there to be a low prevalence of infection in birds near infected farms in endemic regions [14,42], wild birds and mallards in particular [43,44,45] are a tangible risk for the introduction of pathogens in poultry and also for mediating the introduction of pathogens from domestic populations into the avifauna [46].

aMPV transmission is surely facilitated by close contact and the dense population of farmed animals since the duration of the infection and shedding is limited [3]. Therefore, wild hosts may not be as effective a reservoir as reared poultry, which may explain the low prevalence of infection. Conversely, the lack of aMPV-C detection in other species could also be attributed to the small sample size given that mallards accounted for almost half of the sampled birds.

In Italy, aMPV subtype C has not been detected in farmed animals yet, whereas it was identified in wild birds in Northern Italy in 2007 [22]. Specifically, a strain belonging to the European lineage that is phylogenetically close to viruses that were collected from French Muscovy ducks was identified in a Eurasian wigeon. The strain from the present study is closer to Chinese strains and was placed in an intermediate position between the Chinese and French/Italian clusters (Figure 1 and Figure 2). The genetic heterogeneity of the two Italian strains might suggest the presence of different strains in the two populations, but the temporal distance of the detections and the absence of intermediate data prevent any conclusions about the segregation or evolution of the strains. Nonetheless, the migration routes of mallards and wigeons between Central Europe and Eastern regions are similar [47], with mallards reaching more distant areas and are possibly closer to animals carrying the Chinese cluster aMPV-C subtype. However, the paucity of findings and the lack of sequences from other geographic areas prevent the reconstruction of aMPV subtype C history and spreading patterns.

Furthermore, biomolecular assays can only detect active and subclinical infections, thus underestimating viral circulation in the wild bird population. A serological survey may have shown a more detailed picture of their actual exposure to aMPV. Nevertheless, blood sampling is a more invasive procedure and, for welfare reasons, it is often not feasible or is limited to dead birds when compatible with the preservation status of the carcass.

The circulation of various subtypes in the same territory should prompt greater monitoring that preferences species-specific methods rather than those that are subtype-specific. In fact, similar to the hypothesis of an underestimation of aMPV-D circulation due to the use of subtype-specific assays [17], the narrow inclusivity of the most common assays may also contribute to the lack of detection of subtype C. Despite the tropism for ducks [17] of the lineage herein detected, infection in chickens caused by duck aMPV-C was reported in China [25]. This indicates the potential for the spillover of this lineage into farmed animals, where farming conditions could enhance the pathogenicity of duck aMPV-C in a multifactorial picture.

To recognize this eventuality, the adoption of molecular assays with broader specificity should be flanked by serological screening. This could help to promptly identify the early circulation of aMPV-C. However, the presence of aMPV in hosts other than poultry highlights the need for updating the serological tools used in order to screen various bird species and detect circulation of the different subtypes.

## 5. Conclusions

The sampling and screening of thousands of wild animals would not have been possible without synergic action and the dedicated efforts and resources of an authorized and institutional project. Despite the negligible role of wild birds in hosting aMPV-A and B subtypes, the direct identification of subtype C in a wild mallard suggests the importance of close monitoring of both this agent and host. In fact, the biological features of aMPV, such as the short duration of infection and shedding, limit the likelihood of detection, thereby increasing its relevance. On the other hand, the absence of the new subtypes necessitates dedicated studies to investigate their geographic and host range and deepen our understandings of their epidemiology.

## Figures and Tables

**Table 1:** Primers and probes designed for the multiplex real time RT-PCR assays detecting the various aMPV subtypes. Primers and probes were designed using reference sequences (^a^ subtype A Acc. Num. MF093139.1; ^b^ subtype B Acc. Num MN729604.1; ^c^ subtype C Acc. Num. AY579780.1; ^d^ subtype detected in parakeets Acc. Num. MK491499.1; ^e^ subtype detected in gulls Acc. Num. MN175553.1).

Primer/Probe	Sequence 5′→3′	Position
aMPV **A** Forward	CACCCAGGAGCAGCCAACTA	6333–6352 ^a^
aMPV **A** Probe	5′***HEX*** TGCTGGAGTCGCACTTGGTGC 3′***BHQ1***	6355–6375 ^a^
aMPV **A** Reverse	TGTTCGAGCCGTTTGTAATCCTC	6386–6408 ^a^
aMPV **B** Forward	TGGGCAGAAAATGGATCCTTACA	6209–6231 ^b^
aMPV **B** Probe	5′***FAM*** GGCGACTGGAGCAGGAAAGTTTGA 3′***BHQ1***	6301–6324 ^b^
aMPV **B** Reverse	CCATCAACAACTTGCACATACCC	6332–6354 ^b^
aMPV **C** Forward	CAAGGGATCCAGAGGTGAGG	6427–6446 ^c^
aMPV **C** Probe	5′***TAMRA*** CAAGCCCCAGGCCAATGAAG 3′***BHQ2***	6461–6480 ^c^
aMPV **C** Reverse	GAGGTTCCTGCTTGGGTTTG	6487–6506 ^c^
aMPV **PAR-05** Forward	GCGAAACCGATCCAAGACTC	6543–6562 ^d^
aMPV **PAR-05** Probe	5′***CY5*** CACACAAGCAGACCACAACAACAGA 3′***BHQ3***	6595–6619 ^d^
aMPV **PAR-05** Reverse	GAATCTTTGGGGCTTGCTTG	6629–6648 ^d^
aMPV **GuMPV B29** Forward	AAGTTGCGGAGTCAGTGCAA	12240–12259 ^e^
aMPV **GuMPV B29** Probe	5′***FAM*** CAGGGAGGAGCCCTCGTCAA 3′***BHQ1***	12281–12300 ^e^
aMPV **GuMPV B29** Reverse	CGGTGGCACTATGTCGATGT	12326–12345 ^e^

**Table 2:** Number of sampled wild birds and relative species.

Bird	Order	Species	N. of Samples
Mallard	*Anseriformes*	*Anas platyrhynchos*	862 *
Eurasian teal	*Anseriformes*	*Anas crecca*	261
Garganey	*Anseriformes*	*Spatula querquedula*	256
Eurasian wigeon	*Anseriformes*	*Mareca penelope*	230
Northern shoveler	*Anseriformes*	*Spatula clypeata*	70
Eurasian reed warbler	*Passeriformes*	*Acrocephalus scirpaceus*	41
Eurasian blackcap	*Passeriformes*	*Sylvia atricapilla*	41
Gadwall	*Anseriformes*	*Mareca strepera*	37
Cetti’s warbler	*Passeriformes*	*Cettia cetti*	24
Northern pintail	*Anseriformes*	*Anas acuta*	21
Marsh warbler	*Passeriformes*	*Acrocephalus palustris*	17
Great cormorant	*Suliformes*	*Phalacrocorax carbo*	10
Melodious warbler	*Passeriformes*	*Hippolais polyglotta*	8
Common nightingale	*Passeriformes*	*Luscinia megarhynchos*	7
Common kingfisher	*Coraciiformes*	*Alcedo atthis*	6
Great tit	*Passeriformes*	*Parus major*	6
Common blackbird	*Passeriformes*	*Turdus merula*	6
Long-tailed tit	*Passeriformes*	*Aegithalos caudatus*	4
Common moorhen	*Gruiformes*	*Gallinula chloropus*	4
Common pochard	*Anseriformes*	*Aythya ferina*	3
Italian sparrow	*Passeriformes*	*Passer italiae*	3
European robin	*Passeriformes*	*Erithacus rubecula*	2
Common pheasant	*Galliformes*	*Phasianus colchicus*	2
Great reed warbler	*Passeriformes*	*Acrocephalus arundinaceus*	1
Greylag goose	*Anseriformes*	*Anser anser*	1
Cattle egret	*Pelecaniformes*	*Bubulcus ibis*	1
Black woodpecker	*Piciformes*	*Dryocopus martius*	1
Eurasian coot	*Gruiformes*	*Fulica atra*	1
Common chiffchaff	*Passeriformes*	*Phylloscopus collybita*	1
European green woodpecker	*Piciformes*	*Picus viridis*	1
Water rail	*Gruiformes*	*Rallus aquaticus*	1
Common starling	*Passeriformes*	*Sturnus vulgaris*	1
Unknown bird	-	-	2
Total			1932

* Out of 862 mallards, 1 was positive for aMPV-C. All of the remaining individuals were negative for all of the subtypes that were tested.

## Supplementary Materials

The following supporting information can be downloaded at: https://www.mdpi.com/article/10.3390/vetsci9070373/s1, Table S1: Database of avian and human Metapneumovirus sequences of the M gene; Table S2: Database of avian and human Metapneumovirus sequences of the G gene.
