# Supplementary material for: Molecular Survey on A, B, C and New Avian Metapneumovirus (aMPV) Subtypes in Wild Birds of Northern-Central Italy
Source: Vet Sci. 2022 Jul 20;9(7):373. doi: 10.3390/vetsci9070373 (PMC9319881; doi:10.3390/vetsci9070373)
Supplement: Supplementary file 1 [file vetsci-09-00373-s001.zip › vetsci-1742912-supplementary.pdf]

Supplementary Table S1: database of avian and human Metapneumovirus sequences of the M gene downloaded from GenBank and/or previously obtained, with identification, host, accession number, collection year and country.

Virus	*Strain*	*Acc. Num.*	*Country*	*Year*	*Host*	*Identification*
aMPV-C	6264-41	ON457995	Italy	2018	Mallard	aMPV/C/IT/Mallard/6264-41/18
aMPV-C	758/07	OM021855	Italy	2007	Wigeon	aMPV/C/IT/Wigeon/758/07
aMPV-C	470/02	FJ195344	USA	2002	Goose	aMPV/C/USA/Goose/470/02
aMPV-C	36/01	FJ195333.1	USA	2001	Coot	aMPV/C/USA/Coot/36/01
aMPV-C	2a	FJ977568.1	USA	2009	Turkey	aMPV/C/USA/Turkey/2a/97
aMPV-C	144/01	FJ195337.1	USA	2001	Coot	aMPV/C/USA/Coot/144/01
aMPV-C	45/01	FJ195334.1	USA	2001	Coot	aMPV/C/USA/Coot/45/01
aMPV-C	PL/2	EF199772.1	Korea	2007	Pheasant	aMPV/C/Korea/Pheasant/PL-2/07
aMPV-C	PL/1	EF199771.1	Korea	2007	Pheasant	aMPV/C/Korea/Pheasant/PL-1/07
aMPV-C	APV/CO	AY579780.1	USA	1998	Turkey	aMPV/C/USA/Turkey/APV-CO/98
aMPV/C	aMPV/CO	AY590688.1	USA	1996	Turkeys	aMPV/C/USA/aMPV-CO
aMPV-C	Sparrow	AF266674.1	USA	2000	Sparrow	aMPV/C/USA/Sparrow/00
aMPV/C		AF262571.1	USA			aMPV/C/AF262571.1
aMPV/C	Mn1b	AF262569.1	USA			aMPV/C/USA/MN1b
aMPV-C	04	DQ009484.1	USA	2001	Goose	aMPV/C/USA/Goose/04
aMPV/C	Mn1a	AF187149.1	USA			aMPV/C/USA/MN1a
aMPV/C	225/01	FJ195340.1	USA	2001	Goose	aMPV/C/USA/Goose/225/01
aMPV/C	98/01	FJ195336.1	USA	2001	Coot	aMPV/C/USA/Coot/98/01
aMPV/C	56/01	FJ195335.1	USA	2001	Coot	aMPV/C/USA/Coot/56/01
aMPV-C	35/01	FJ195332.1	USA	2001	Coot	aMPV/C/USA/Coot/35/01
aMPV/C	MN6	AF298636.1	USA	1999	Turkey	aMPV/C/USA/Turkey/MN6/99
aMPV/C	MN7	AF298635.1	USA	1999	Turkey	aMPV/C/USA/Turkey/MN7/99
aMPV/C	MN9	AF298633.1	USA	1999	Turkey	aMPV/C/USA/Turkey/MN9/99
aMPV/C	146/01	FJ195338.1	USA	2001	Coot	aMPV/C/USA/Coot/146/01
aMPV/C	MN4a	AF298641.1	USA	1999	Turkey	aMPV/C/USA/turkey/MN4A/99
aMPV/C	MN4C	AF298640.1	USA	1999	Turkey	aMPV/C/USA/Turkey/MN4C/99
aMPV/C	MN11	AF298637.1	USA	1999	Turkey	aMPV/C/USA/Turkey/MN11/99

aMPV/C	MN8	AF298634.1	USA	1999	Turkey	aMPV/C/USA/Turkey/MN8/99
aMPV-C	Duck	AF266673	USA	2000	Duck	aMPV/C/USA/Duck/00
aMPV/C	MN2a	AF187151.1	USA	1997	Turkey	aMPV/C/USA/Turkey/MN2A/97
aMPV/C	228/01	FJ195341.1	USA	2001	Goose	aMPV/C/USA/Goose/228/01
aMPV/C	MN4b	AF298639.1	USA	1999	Turkey	aMPV/C/USA/Turkey/MN4B/99
aMPV-C	Goose	AF266675.1	USA	2000	Goose	aMPV/C/USA/Goose/00
aMPV/C	Georgia/02	FJ195345.1	USA	2002	Goose	aMPV/C/USA/Goose/02
aMPV/C	322/01	FJ195343.1	USA	2001	Goose	aMPV/C/USA/Goose/322/01
aMPV/C	148/01	FJ195339.1	USA	2001	Coot	aMPV/C/USA/Coot/148/01
aMPV/C	MN-12	AF368171.1	USA		Duck	aMPV/C/USA/Duck/MN-12
aMPV-C	239	FJ195342.1	USA	2001	Goose	aMPV/C/USA/Goose/239/01
aMPV/C	32/01	FJ195331.1	USA	2001	Coot	aMPV/C/USA/Coot/32/01
aMPV/C	24/01	FJ195329.1	USA	2001	Coot	aMPV/C/USA/Coot/24/01
aMPV/C	MN10	AF298638.1	USA	1999	Turkey	aMPV/C/USA/Turkey/MN10/99
aMPV/C	26/01	FJ195330.1	USA	2001	Coot	aMPV/C/USA/Coot/26/01
aMPV-C	99178	HG934338.1	France	1999	Duck	aMPV/C/FR/MuscovyDuck/99178/99
aMPV-C	S01	KF364615	China	2001	Duck	aMPV/C/China/Duck/S01/11
aMPV-C	GDY	KC915036.1	China	2011	Duck	aMPV/C/China/GDY/11
aMPV-C	JC	JX422020.1	China	2012	Chicken	aMPV/C/China/Ck/JC/12
aMPV/C	MN5	AF298651.1	USA	1999	Turkey	aMPV/C/USA/Turkey/MN5/99
aMPV-A	8544	DQ666911.1	Italy	2006		aMPV/A/IT/8544/06
aMPV-B	Vr240	(Courtesy of Prof. Catelli)	Italy	1987	Turkey	aMPV/B/IT/Ty/Vr240/87
aMPV-D	85035	HG934339.1	France	1985	Turkey	aMPV/D/FR/Ty/85035/85
hMPV-A1	00/1	AF371337.2	Netherlands	2001	Human	hMPV/A1/NL/Human/00-1/01
hMPV-A2	NL/00/17	FJ168779.1	Netherlands	2000	Human	hMPV/A2/NL/Human/NL-00-17/00
hMPV-B1	NL/1/99	AY525843.1	Netherlands	1999	Human	hMPV/B1/NL/Human/NL-1-99/99
hMPV-B2	NL/94/01	FJ168778.1	Netherlands	1994	Human	hMPV/B2/NL/Human/NL-94-01/94
GuMPV	B29	MN175553.1	Canada	2015	Great black beaked gull	GuMPV/C/USA/Duck/MN-12
PAR-MPV	PAR-05	MK491499.1	USA	2018	Monk Parakeet	PAR-MPV/USA/MonkParakeet/PAR-05/2018

Supplementary Table S2: database of avian and human Metapneumovirus sequences of the G gene downloaded from GenBank and/or previously obtained, with identification, host, accession number, collection year and country.

Virus	*Strain*	*Acc. Num.*	*Country*	*Year*	*Host*	*Identification*
aMPV-C	6264-41	ON457994	Italy	2018	Mallard	aMPV/C/IT/Mallard/6264-41/18
aMPV-C	758/07	OM021856	Italy	2007	Wigeon	aMPV/C/IT/Wigeon/758/07
aMPV-C	99178	HG934338.1	France	1999	Duck	aMPV/C/FR/MuscovyDuck/99178/99
aMPV-C	99350	AJ811992.1	France	1999	Muscovy duck	aMPV/C/FR/MuscovyDuck/99350/99
aMPV-C	00094	AJ811993.1	France	1999	White Pekin duck	aMPV/C/FR/ WhitePekinDuck/00094/99
aMPV-C	GDY	KC915036.1	China	2011	Duck	aMPV/C/China/GDY/11
aMPV-C	S01	KF364615.2	China	2011	Muscovy duck	aMPV/C/China/MuscovyDuck/S01/11
aMPV-C	15a/01	GU126687.1	USA	2001	Canada goose	aMPV/C/USA/CanadaGoose/15a/01
aMPV-C	Mn-2a	AY590693.1	USA	1997	Turkey	aMPV/C/USA/Turkey/MN2A/97
aMPV-C	Mn1a	AY590692.1	USA			aMPV/C/USA/MN1a
aMPV-C	PL/2	EF199772.1	Korea	2007	Pheasant	aMPV/C/Korea/Pheasant/PL-2/07
aMPV-C	PL/1	EF199771.1	Korea	2007	Pheasant	aMPV/C/Korea/Pheasant/PL-1/07
aMPV-C	Colorado	AY590691.1	USA		Turkey	aMPV/C/USA/Turkey/Colorado
aMPV/C	aMPV/CO	AY590688.1	USA	1996	Turkeys	aMPV/C/USA/aMPV-CO
aMPV-A	8544	DQ666911.1	Italy	2006		aMPV/A/IT/8544/06
aMPV-B	657	MN729604	Hungary	1989	Turkey	aMPV/B/Hungary/Turkey/657-4/89
aMPV-D	85035	HG934339.1	France	1985	Turkey	aMPV/D/FR/Ty/85035/85
hMPV-A1	00/1	AF371337.2	Netherlands	2001	Human	hMPV/A1/NL/Human/00-1/01
hMPV-A2	NL/00/17	FJ168779.1	Netherlands	2000	Human	hMPV/A2/NL/Human/NL-00-17/00
hMPV-B1	NL/1/99	AY525843.1	Netherlands	1999	Human	hMPV/B1/NL/Human/NL-1-99/99
hMPV-B2	NL/94/01	FJ168778.1	Netherlands	1994	Human	hMPV/B2/NL/Human/NL-94-01/94
PAR-MPV	PAR-05	MK491499.1	USA	2018	Monk Parakeet	aMPV/PAR-MPV/USA/MonkParakeet/PAR-05/2018
GuMPV	B29	MN175553.1	Canada	2015	Great black beaked gull	aMPV/GuMPV/Canada/ GreatBlackBeakedGull/B29/15
